# Global Prevalence of Overactive Bladder: A Systematic Review and Meta-analysis

**DOI:** 10.1007/s00192-024-06029-2

**Published:** 2025-02-14

**Authors:** Lin Zhang, Nian Cai, Li Mo, Xiaofang Tian, Hongcen Liu, Bohai Yu

**Affiliations:** Shenzhen Hospital (Futian) of Guangzhou University of Chinese Medicine, Shenzhen City, Guangdong Province China

**Keywords:** Prevalence, Overactive bladder (OAB), Global, Meta-analysis, Epidemiology

## Abstract

**Introduction and Hypothesis:**

This study aims to systematically estimate the global prevalence of overactive bladder (OAB), identify demographic and regional factors contributing to prevalence variations, and assess trends in prevalence over the past two decades.

**Methods:**

This cross-sectional study was conducted according to the PRISMA (Preferred Reporting Items for Systematic Reviews and Meta-Analyses) guideline. From inception to April 2024, computerized searches for OAB prevalence-related literature were conducted on PubMed, Embase, Web of Science, and Cochrane. Studies of OAB prevalence in the general population were included. Two independent researchers conducted the screening, data extraction, and quality assessment of the included studies.

**Results:**

A total of 53 studies, encompassing 610,438 participants, were ultimately included in the analysis. The meta-analysis determined the global prevalence of OAB to be 20% (95% CI 0.18–0.21). Over the past 20 years, there has been an increase in OAB prevalence, rising from 18.1% (95% CI 0.13–0.23) to 23.9% (95% CI 0.19–0.29). Among women, the prevalence of OAB was 21.9% (95% CI 0.20–0.24), indicating higher rates compared to men (OR = 16.1, 95% CI 0.15–0.18). The study also found higher prevalence rates among overweight and obese individuals (OR = 18.6, 95% CI 0.13–0.24) and those aged 60 years and above (OR = 28.3, 95% CI 0.24–0.33). Middle-income countries exhibited higher prevalence rates compared to high-income countries.

**Conclusions:**

The study highlights higher risks of OAB among obese individuals, women, and the elderly. OAB prevalence has shown an increasing trend over the past 20 years.

## Introduction

Overactive bladder (OAB) is a common and significant lower urinary tract disorder marked by a strong urge to urinate. It frequently includes symptoms such as increased urinary frequency, nocturia, and urgency incontinence, and occurs in the absence of urinary tract infections or other identifiable pathologies [1]. The pathogenesis of OAB is multifaceted, involving the overactivity of the detrusor muscle and dysregulation within the central nervous system [2]. Modern lifestyle changes, such as increased stress and sedentary habits, have contributed to a rise in OAB prevalence, imposing a growing burden on both individual health and healthcare systems [3, 4].

OAB significantly deteriorates patients’ quality of life (QOL) and imposes substantial economic burdens [5]. In 2020, the total prevalence of OAB across eight major countries—the United States, five European nations (France, Germany, Italy, Spain, and the United Kingdom), Japan, and China—was approximately 363 million cases. This number is expected to rise to 401.6 million by 2030 [6]. This condition represents a significant public health issue, affecting one in seven women and a comparable proportion of men in the US. Beyond the personal and individual burdens it imposes, OAB contributes to substantial annual societal expenses, underscoring the imperative for effective treatment strategies [7, 8].

Despite the extensive impact of OAB, its prevalence varies widely among countries and regions. Large-scale epidemiologic studies report prevalence rates ranging from 8.8% in China to 23.2% in the United States [9, 10]. These variations may stem from differences in study design, population demographics, sampling methods, and sample sizes. While many studies have investigated the prevalence of OAB in diverse regions and populations, the results frequently exhibit inconsistency. This inconsistency highlights the need for a comprehensive synthesis and evaluation of available data through systematic literature reviews and meta-analyses.

To address this gap, the current study utilizes a systematic review and meta-analysis to offer a global estimation of OAB prevalence. By systematically reviewing and analyzing data from diverse studies, this research aims to offer a more accurate and comprehensive understanding of OAB prevalence, elucidating trends and regional differences. Such insights are critical for the development of effective public health policies and clinical interventions aimed at mitigating the impact of OAB.

## Method

### Search Strategy

The study adheres to both the PRISMA (Preferred Reporting Items for Systematic Reviews and Meta-Analyses) [11] and AMSTAR (Assessing the Methodological Quality of Systematic Reviews) [12] guidelines. Moreover, it has been successfully registered with PROSPERO (International Prospective Register of Systematic Reviews, CRD42024534398).

We systematically searched all published literature on the prevalence of OAB from the inception of each database until April 2024, encompassing PubMed, Embase, Web of Science, and Cochrane. A comprehensive set of search terms related to overactive bladder (e.g., “overactive bladder” or “urinary bladder, overactive”) and prevalence (e.g., “prevalence” or “epidemiology”) was employed. No restrictions were applied regarding study design, sample size, country, or language. The comprehensive search strategy for each database is outlined in Appendix Table 3.

### Inclusion and Exclusion Criteria

Studies meeting the following criteria were considered for inclusion: (1) original survey studies based on representative samples; (2) participants from all age groups and both genders; and (3) reported the number of individuals with overactive bladder along with prevalence estimates. Conversely, studies were excluded if they: (1) constituted non-original research, such as conference abstracts, case reports, and review papers; (2) lacked relevant data; or (3) focused on particular populations, such as female military personnel, patients with hypertension or diabetes, or those seeking urological care.

### Study Selection

To ensure consistency in prevalence rates and minimize data bias, we recalculated weighted prevalence rates based on the original prevalence rates across all included studies. In cases where multiple articles reported data from the same survey (duplicate literature), only the study with the largest combined results or sample size was retained.

Our study selection process involved multiple steps. Initially, duplicate records were removed from various databases. Following that, two researchers autonomously scrutinized the titles and abstracts of the screened articles, acquiring the complete texts of potentially suitable articles for thorough evaluation against our inclusion criteria. Ultimately, the references of articles included in the full-text screening stage were thoroughly examined to mitigate any potential bias resulting from omission. Any discrepancies were resolved through consensus or consultation with a third party.

### Data Extraction

Key data from the enclosed articles, including titles, authors, publication years, investigation years, study locations (both country and region), diagnostic tools, sample sizes, age ranges, body mass indexes (BMIs), and prevalence rates, were independently extracted by two researchers.

Study regions were delineated in accordance with World Health Organization (WHO) criteria, encompassing the African region, Region of the Americas, Eastern Mediterranean region, European region, and Western Pacific region. Furthermore, countries were stratified according to World Bank (WB) criteria as high-income, middle-income, or low-income.

### Quality Assessment

Two investigators independently assessed the risk of bias in the incorporated studies, with the outcomes subsequently cross-validated for coherence. The evaluation of study quality employed an 11-item checklist endorsed by the Agency for Healthcare Research and Quality (AHRQ) [13]. The dimensions assessed encompassed the source of information, nadir criteria, response rate, data collection methods, and outcome assessment. Each criterion received a score of 0 for a "no" or "unclear" response and 1 for a "yes" response, resulting in total scores ranging from 0 to 11. Higher scores correlated with a reduced risk of bias, indicative of superior study quality. Remarkably, scores falling within the range of 0 to 3 were indicative of low quality, whereas those between 4 and 7 denoted moderate quality, and scores from 8 to 11 underscored high quality.

### Statistical Analysis

The meta-analysis was conducted using Stata 15.0 software. The assessment of heterogeneity among the studies was conducted utilizing Cochran’s Q test and the *I*^*2*^ index. A *P* value of < 0.05 in Cochran’s Q test indicates significant heterogeneity. An *I*^*2*^ index of < 25% signifies low heterogeneity, 26–50% represents moderate heterogeneity, and > 50% indicates high heterogeneity. In instances of substantial heterogeneity, a random-effects model (the DerSimonian and Laird method) was employed; conversely, a fixed-effects model was utilized to determine the overall prevalence of OAB and its corresponding 95% confidence intervals. Meta-regression analyses were subsequently conducted to ascertain moderators elucidating the heterogeneity of prevalence. The stability of the results was assessed through sensitivity analysis using the one-by-one exclusion method. Additionally, publication bias was evaluated using funnel plots and Egger’s test.

## Result

### Study Selection and Characteristics

A preliminary search yielded 8333 articles. Utilizing the predefined inclusion and exclusion criteria, the subsequent entries were omitted: (1) 6733 articles were not relevant; (2) 1539 articles were duplicates; and (3) 52 articles without sufficient information. Ultimately, 53 articles were included for further analysis [14–30] (PRISMA flow diagram). Furthermore, the bibliographies of the included articles underwent thorough scrutiny, revealing no additional relevant articles.

Table 1 provides a comprehensive overview of the features of the 53 encompassed articles, all of which were cross-sectional studies. All included studies had defined inclusion and exclusion criteria, which generally excluded populations with urinary system symptoms or pelvic organ diseases (either self-reported or clinically diagnosed), as well as individuals undergoing related pharmacological treatments. The sample sizes of these studies varied significantly, ranging from 280 to 226,867 participants, with a total of 610,438 individuals, including 52,064 patients diagnosed with OAB. The reported prevalence of OAB exhibited considerable variation, from 2.1% to 67.0%, with 36 studies (68.0%) providing prevalence data for gender subgroups. Additionally, the majority of these articles were published in the past decade (*n* = 33, 62.3%). Over half of the studies were conducted in the Western Pacific region (56.6%), and the vast majority were carried out in upper-middle-income countries (90.5%). The studies utilized various questionnaires to assess OAB, with 12 (22.6%) articles not specifying the assessment questionnaire used.

**Table 1:** Main characteristics of included studies for meta-analysis on the prevalence of OAB

Author (ref)	Country/ region	Date collection		Age range	Simple size	OAB cases	Prevalence OAB (%)			Diagnostic criteria	AHRQ
							Male	Female	All		
Daily AM et al. 2019 [14]	USA	2017		> 18	6562	1059	–	16.1%	16.1%	ICIQ‐OAB	9
Santander J et al. 2022 [15]	Colombian	–		> 18	1060	336	24.15%	39.25%	31.7%	ICIQ‐OAB	8
Ikeda Y et al. 2011 [16]	Japan	2003		> 70	833	153	17.6%	19.1%	18.4%	IPSS	9
Chae J et al. 2018 [17]	Korea	2014–2015		18–80	812	157	–	19.3%	19.3%	OABSS	7
Liang Y et al. 2022 [18]	China	2019–2022		18–22	13,083	788	4.7%	6.7%	6.0%	OABSS	9
Wang Y et al. 2011 [19]	China	2009–2010		> 18	14,844	1268	5.9%	6.0%	6.0%	OABSS	7
Cheung WW et al. 2009 [20]	USA	–		> 16	311	160	60.5%	48.3%	51.4%	OAB–V8	6
Temml C et al. 2005 [21]	Austria	–		20–91	2418	327	10.18%	16.8%	13.5%	–	6
Safarinejad MR et al. 2009 [22]	Iran	2004–2006		15–55	7806	1421	–	18.2%	18.2%	–	9
Wu JW et al. 2016 [23]	China	2013–2014		> 40	1061	145	13,2%	14.1%	13.7%	OABSS	9
Lee YS et al. 2011 [24]	Korea	2006		≥ 18	2000	244	10.0%	14.3%	12.2%	IPSS	7
Sarici H et al. 2016 [25]	Türkiye	2013		20–50	1636	338	–	20.7%	20.7%	–	9
AI Edwan G et al. 2021 [26]	Middle East*	2018		≥ 40	2297	1235	–	53.8%	53.8%	OAB–V8	9
Chuang YC et al. 2019 [27]	China, Korea	–		≥ 40	8284	1726	19.5%	22.1%	20.8%	OABSS	6
Moorthy P et al. 2004 [28]	Asian*	1998		≥ 18	2369	709	29.9%	–	29.9%	–	9
Qudah S et al. 2024 [29]	Jordan		2021–2022	–	940	258	22.0%	32.4%	27.4%	OABSS	8
Kim MK et al. 2022 [31]	Korea		2022	≥ 19	2000	192	10.3%	9.0%	9.6%	OABSS	6
Plata M et al. 2019 [32]	Colombian		2015	≥ 18	1060	458	–	–	43.2%	ICIQ-OAB	9
Coyne KS et al. 2013 [10]	USA		2010	18–70	10,000	2320	16.4%	30.0%	23.2%	OAB-POLL	9
Coyne KS et al. 2011 [33]	USA		2008	–	2000	684	26.1%	41.2%	34.2%	–	7
Yang CF et al. 2022 [34]	China		–	40–65	970	648	–	67.0%	67.0%	ICIQ-OAB	6
Ninomiya S et al. 2018 [35]	Japan		–	20–79	4804	387	–	8.1%	8.1%	OABSS	6
Zhang W et al. 2006 [36]	China		2002	≥ 20	4684	377	–	8.0%	8.0%	–	7
de Boer TA et al. 2011 [37]	Netherlands		–	45–85	1397	677	–	48.6%	48.6%	–	6
Xing D et al. 2020 [38]	China		2018	5–14	10,133	913	8.86%	9.18%	9.01%	–	7
Wen JG et al. 2014 [39]	China		2010–2011	≥ 40	9805	209	2.7%	1.9%	2.1%	OABSS	8
Chiu AF et al. 2012 [40]	China		2010	≥ 40	1011	195	19.9%	18.7%	19.8%	OABSS	9
Sexton CC et al. 2011 [41]	USA		2005	≥ 65	5362	2354	40.4%	46.9%	43.9%	OAB-q SF	8
Chen GD et al. 2003 [42]	China		1999	≥ 20	1247	232	–	18.6%	18.6%	The Bristol Female Urinary Tract Symptoms Questionnaire	7
Stewart WF et al. 2003 [4]	USA		–	≥ 18	5204	857	16.0%	16.9%	16.5%	–	8
Rashid S et al. 2021 [43]	Pakistan		2020	35–60	1058	289	25.7%	31.3%	27.4%	OABSS	9
Chung JM et al. 2009 [44]	Korea		2006	5–13	16,516	2740	–	–	16.59%	–	7
Kim SY et al. 2017 [45]	Korea		2012	19–107	94,554	3610	2.9%	–	2.9%	OABSS	7
Kim SY et al. 2017 [46]	Korea		2012	19–107	107,950	6814	–	5.2%	5.2%	OABSS	7
Jo JK et al. 2012 [47]	Korea		2010	> 40	926	130	12.2%	15.5%	14.1%	OABSS	7
Ru J et al. 2022 [48]	China		2020	6–15	2333	141	6.0%	–	6.0%	OABSS	7
Salcedo FL et al. 2013 [49]	Spain		2010	≥ 18	1004	119	–	11.8%	11.8%	CACV	7
Sut HK et al. 2012 [50]	Türkiye		2010	30–65	280	109	–	38.9%	38.9%	OAB-V8	9
Ng SC et al. 2017 [51]	China		2012	> 40	1469	487	–	33.1%	33.1%	OABSS	7
Funada S et al. 2018 [52]	Japan		2008–2009	30–74	4645	549	15.3%	10.1%	11.8%	OABSS	9
Homma Y et al. 2005 [53]	Japan		–	> 40	4570	556	14.0%	11.0%	12.4%	The Japan Neurogenic Bladder Society Committee	6
Abreu GE et al. 2018 [54]	Brazil		2017	> 20	516	79	–	15.3%	15.3%	ICIQ-OAB	9
Omae K et al. 2019 [55]	Japan		2017	> 75	314	88	–	–	28.0%	OABSS	9
Ishimaru T et al. 2020 [56]	Japan		2014–2019	≥ 65	4782	784	–	–	16.4%	OABSS	7
Kim SK et al. 2021 [57]	Korea		2012	> 19	226,867	12,303	–	–	5.4%	OABSS	7
Yoo ES et al. 2011 [58]	Korea		2010	≥ 30	2000	458	19.0%	26.8%	22.9%	OABSS	9
Tikkinen KA et al. 2007 [59]	Finland		2003–2004	18–79	3727	295	6.5%	9.3%	8.0%	Danish Prostatic Symptom Score	9
Kim DY et al. 2020 [60]	Korea		–	≥ 18	1490	205	–	10.3%	10.3%	OABSS	8
An F et al. 2016 [9]	China		2012	18–97	2161	191	–	8.8%	8.8%	OABSS	9
Yu HJ et al. 2006 [61]	China		2000–2005	≥ 30	1827	332	17.6%	18.7%	16.9%	–	9
Teloken C et al. 2006 [62]	Brazil		2003–2004	15–55	848	160	14.0%	23.2%	18.9%	the King’s Health Questionnaire	9
Dávila HA et al. 2010 [63]	Venezuela		2003–2007	18–75	3407	716	13.7%	25.6%	21.0%	OAB-V8	7
Sheikh MA et al. 2022 [30]	Pakistan		2017–2018	14–85	1291	82	2.4%	10.2%	6.4%	OABSS	7

Middle East*: Jordan, Egypt, Algeria, Lebanon. Asian*: China, India, Indonesia, Korea, Malaysia, Pakistan, Philippines, Singapore, Taiwan, Thailand

*IPSS*, International Prostate Symptom Score

*OABSS*, Overactive Bladder Symptom Score

*OAB-V8*, Overactive Bladder-Validated 8-question Screener

*CATI*, Computer-assisted Telephone Interview Questionnaire

*ICIQ-OAB*, International Consultation on Incontinence Questionnaire-Overactive Bladder

*AHRQ*, Agency for Healthcare Research and Quality

### Diagnosis of OAB

Table 1 enumerates the measurement instruments utilized to evaluate OAB in the included studies. Stratified analysis revealed that the prevalence rates across different measurement tools ranged from 8% (95% CI 0.07–0.09) to 44% (95% CI 0.43–0.45), demonstrating substantial heterogeneity (*I*^2^ > 90%, *P* < 0.001) (Appendix Fig. 5). The OABSS questionnaire was utilized for quantitative assessment in 24 articles (45.3%). Devised by Blaivas et al., the OABSS quantifies symptoms of OAB into a unified score, incorporating four inquiries: daytime frequency, nighttime frequency, urgency, and urgency incontinence. Patients assess these symptoms, assigning maximum scores of 2, 3, 5, and 5, respectively. The cumulative score ranges from 0 to 15, with higher scores signifying more pronounced OAB.

Four studies used the 8-item Overactive Bladder Questionnaire (OAB-V8) for assessment. The creators of the OAB-V8 developed a logistic regression model to predict OAB diagnosis based on the recorded scores. They found that after a 2-point increase in male patients’ scores, gender ceased to be a significant predictor. However, the studies showed that this did not adequately differentiate the gender-specific responses to the OAB-V8 or the symptom distress [64, 65].

Five investigations employed the Overactive Bladder Module of the International Consultation on Incontinence Questionnaire (ICIQ-OAB). This instrument is appropriate for global populations and facilitates the comparison of outcomes across diverse environments [66]. In 2017, the International Consortium for Health Outcomes Measurement (ICHOM) developed the OAB-q SF, a concise questionnaire designed to evaluate symptom burden and quality of life in individuals with OAB [67]. One study validated its reliability and effectiveness. Another study used the OAB-POLL questionnaire, confirming that OAB symptoms negatively impact physical and occupational functioning [10].

Additionally, two studies used the International Prostate Symptom Score (IPSS) questionnaire for quantitative evaluation. The IPSS is a universally acknowledged instrument for gauging the severity of lower urinary tract symptoms in patients with prostatic hypertrophy, encompassing seven symptom-related items and one item pertaining to quality of life. It is commonly used to evaluate men with lower urinary tract symptoms (LUTS) or benign prostatic hyperplasia (BPH) [53, 68].

Other questionnaires included the Spanish version of the Bladder Control Self-Assessment Questionnaire (CACV), the Dutch King’s Health Questionnaire, the Danish Prostate Symptom Score, the Bristol Female Lower Urinary Tract Symptoms Questionnaire, and a specific questionnaire developed by the Japanese Neurogenic Bladder Society Committee. Each of these was used in a single study.

### Quality Assessment of the Literature

All included studies received a quality score of ≥ 5. Detailed quality assessments are provided in Appendix Table 4. Two researchers scored the studies based on the 11 criteria recommended by AHRQ. Of the assessed articles, 18 were deemed of moderate quality, while 35 were classified as high quality. No studies were classified as low quality.

### Assessment of OAB Prevalence

**Fig. 1:**
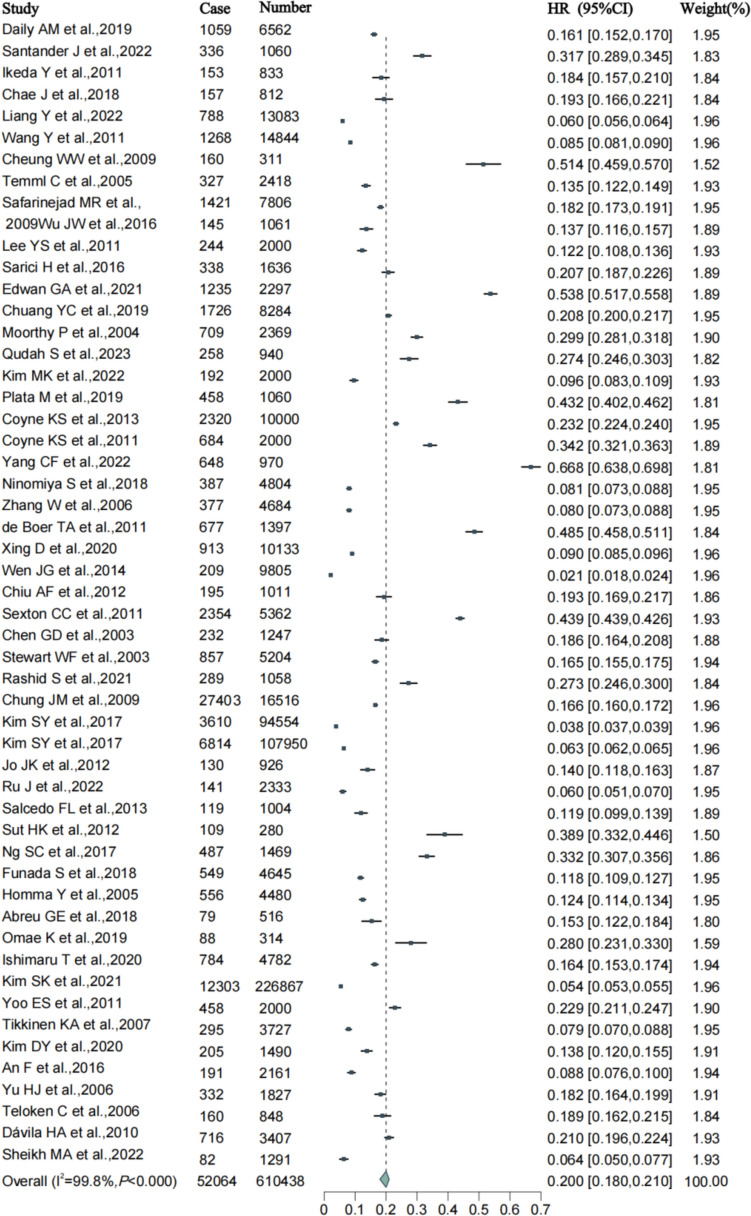
Forest plot of meta-analysis on the prevalence of OAB

The random-effects meta-analysis revealed that the global prevalence of OAB is 20% (95% CI 0.18–0.21) (Fig. 1). Among women, the prevalence is 21.9% (95% CI 0.20–0.24), while among men, it is 16.1% (95% CI 0.15–0.18) (Table 2).

**Table 2:** Global prevalence of OAB using random-effects meta-analysis and subgroup meta-analysis

Variable	No. of articles	No. of participants	No. of cases	Prevalence (95%CI)	*I*^*2*^,%	*P* value	Egger test	Subgroup difference
Sex								–
Overall	52	595,562	50,797	19.5(18.3,20.6)	99.7	< 0.001	< 0.001	
Male	35	256,470	16,744	16.1(14.6,17.6)	99.7	< 0.001		
Female	49	339,092	34,053	21.9(20.1,23.7)	99.5	< 0.001		
BMI								0.53
Overall	28	43,939	3355	14.1(12.3,15.9)	98.3	< 0.001	< 0.001	
< 25	10	25,492	1712	11.7(9.1,14.2)	98.4	< 0.001		
25–29.9	9	14,100	1154	16.1(11.9,20.3)	98.8	< 0.001		
≥ 30	9	4347	489	18.6(12.8,24.3)	97.7	< 0.001		
Publish period								0.71
Overall	53	610,438	52,064	19.9(18.5,21.4)	99.8	< 0.001	< 0.001	
2000–2005	5	15,718	2681	18.1(13.1,23.2)	98.6	< 0.001		
2006–2010	8	39,126	6201	19.3(15.1,23.5)	99.2	< 0.001		
2011–2015	13	51,462	8920	22.8(16.0,29.7)	99.8	< 0.001		
2016–2020	17	252,233	17,990	16.5(14.5,18.6)	99.7	< 0.001		
2021–2024	10	251,899	16,272	23.9(18.6,29.1)	99.8	< 0.001		
Age								0.07
Overall	36	298,182	20,818	18.0(16.5,19.6)	99.7	< 0.001	< 0.001	
< 18	2	25,649	3653	13.3(4.9,21.8)	99.8	< 0.001		
18–39	8	82,297	2879	11.1(8.1,14.1)	99.3	< 0.001		
40–59	11	104,741	4047	13.6(11.3,15.8)	99.2	< 0.001		
≥ 60	15	85,495	10,239	28.3(23.8,32.8)	99.3	< 0.001		
WHO region								0.02*
Overall	52	608,069	51,355	19.7(1.83,2.12)	99.8	< 0.001	< 0.001	
AFR	3	6644	2209	34.1(12.6,55.5)	99.7	< 0.001		
AMR	10	32,923	8467	29.3(22.6,36.0)	99.5	< 0.001		
EMR	5	12,071	2239	21.9(14.5,29.4)	98.9	< 0.001		
EUR	4	8,546	1418	20.4(7.8,33.0)	99.6	< 0.001		
WPR	30	547,885	37,022	14.7(13.4,16.0)	99.7	< 0.001		
WB region								0.45
Overall	53	604,662	50,639	20.3(18.9,21.7)	99.8	< 0.001	< 0.001	
HIC	27	515,038	38,633	18.2(16.5,19.9)	99.8	< 0.001		
UMIC	21	77,368	9283	21.6(18.1,25.0)	99.7	< 0.001		
LMIC	5	12,256	2723	27.4(14.4,40.4)	99.6	< 0.001		

*AFR*, African region; *AMR*, Region of the Americas; *EMR*, Eastern Mediterranean region; *EUR*, European region; *WPR*, Western Pacific region; *HIC*, High-income countries; *UMIC*, Upper- and middle-income countries; *LMIC*, Low- and middle-income countries; *BMI*, Body mass index; *WB*, World Bank; *WHO*, World Health Organization

### Subgroup Analysis

**Fig. 2:**
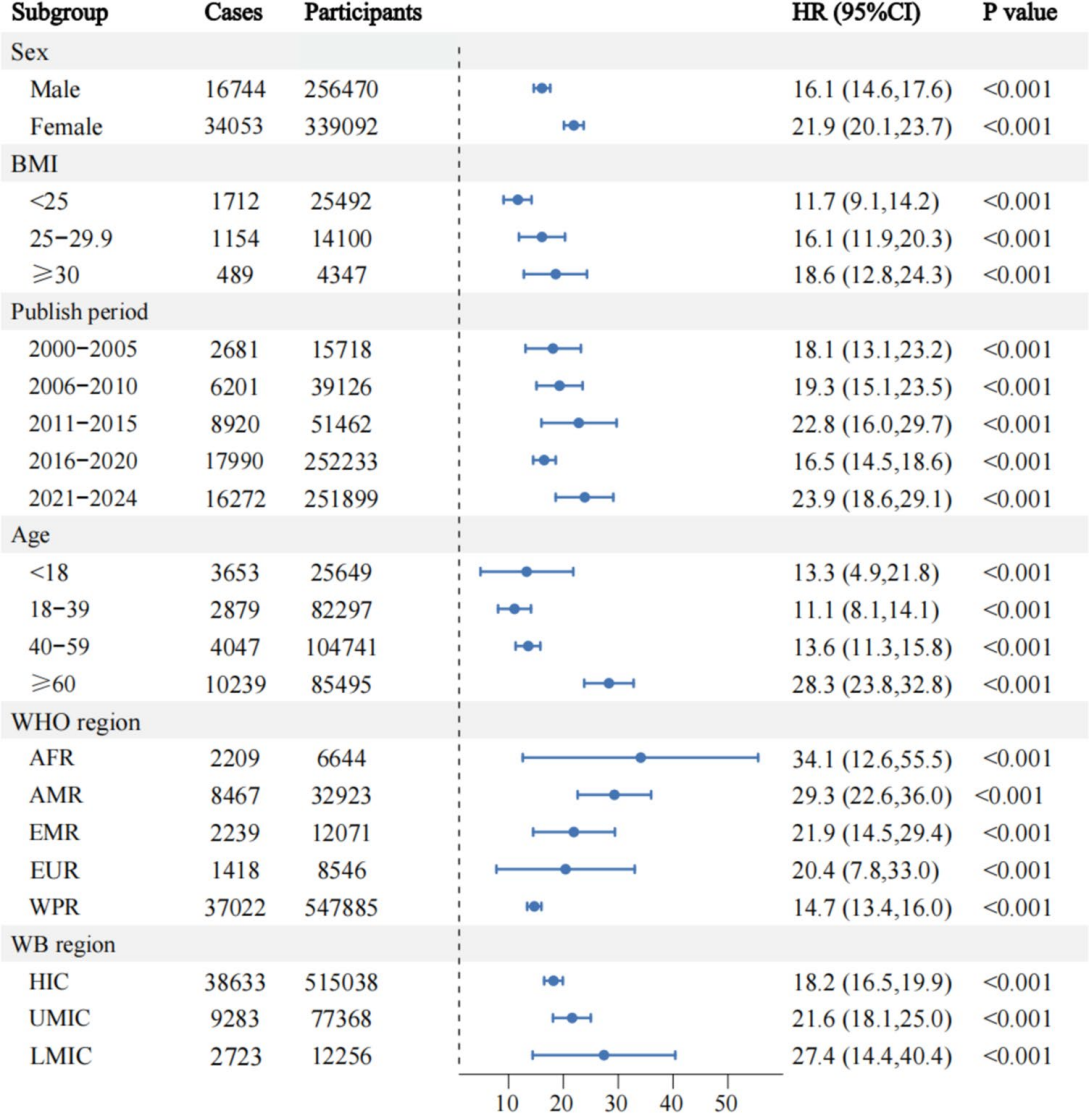
Forest plot of subgroup meta-analysis on the prevalence of OAB

Owing to significant heterogeneity among the studies, we conducted subgroup analyses based on age, gender, BMI, WB region, publication year, and WHO region to identify potential sources of heterogeneity. Meta-regression showed that OAB prevalence is associated with WHO regions (*P* = 0.02). The prevalence rates for AFR, AMR, EMR, EUR, and WPR were 34.1% (95% CI 0.13–0.56), 29.3% (95% CI 0.23–0.36), 21.9% (95% CI 0.15–0.29), 20.4% (95% CI 0.08–0.33), and 14.7% (95% CI 0.13–0.16), respectively (Fig. 2).

Our investigation also revealed that the prevalence among individuals aged 60 years and older was higher than that of other age cohorts, standing at 28.3% (95% CI 0.24–0.33). However, there were no statistically significant differences observed across the various age groups (*P* = 0.07). Women had a higher OAB prevalence than men, at 21.9% (95% CI 0.20–0.24). The incidence among overweight and obese individuals was elevated compared to their normal-weight counterparts, with rates of 16.1% (95% CI 0.12–0.20) and 18.6% (95% CI 0.13–0.24), respectively. Nevertheless, there were no statistically significant disparities across BMI classifications (*P* = 0.53) (Table 2).

Additionally, the prevalence in LMIC countries was higher than in UMIC and HIC countries, at 27.4% (95% CI 0.14–0.40), but without statistical significance (*P* = 0.45). Over time, the global prevalence of OAB has generally increased, rising from 18.1% (95% CI 0.13–0.23) in 2000–2005 to 23.9% (95% CI 0.19–0.29) in 2021–2024, though publication year differences were not statistically significant (*P* = 0.71). Detailed information is provided in Table 2.

### Sensitivity Analysis and Publication Bias

After performing the leave-one-out analysis, the worldwide prevalence of OAB was determined to span from 18 to 21% (Appendix Fig. 4). These outcomes correspond with the general prevalence, underscoring the robustness and dependability of the meta-analysis results. Visual scrutiny of the funnel plot and application of Egger’s test were utilized to evaluate publication bias. The conspicuously asymmetrical funnel plot indicates potential publication bias (Appendix Fig. 3), a deduction corroborated by the findings of Egger’s test (Table 2).

## Discussion

This comprehensive study, adhering to stringent inclusion and exclusion criteria, encompasses 53 epidemiological investigations into OAB. Conducted between 2000 and 2024, these studies span 24 countries across six WHO regions. By means of a systematic review and meta-analysis encompassing these 53 studies, we approximated the worldwide prevalence of OAB to be 20%. Over the past two decades, there has been a discernible upward trend in this prevalence. However, it is essential to acknowledge that the actual prevalence might surpass our estimate, as many patients, due to psychological barriers, may refrain from seeking medical treatment, often perceiving OAB symptoms as an aspect of aging rather than a medical condition [69].

We speculate that the escalating prevalence of OAB could be attributed to factors such as an aging population, the surge in obesity, and the enhanced status of women. These factors correlate positively with recognized risk factors for OAB, including age, obesity, and gender [70, 71].

As indicated by numerous studies [72, 73], the prevalence of overactive bladder (OAB) is higher in women than in men, which may be attributed to unique physiological and anatomical characteristics of women, making them more prone to urinary tract infections (UTIs) [74]. Individuals afflicted with UTIs face an elevated susceptibility to the onset of overactive bladder syndrome. UTIs can lead to bladder irritation and dysfunction, potentially triggering the onset of OAB. During gestation, the enlarging uterus exerts pressure on the bladder, reducing its capacity and altering its storage function, which may lead to abnormal urination patterns and detrusor overactivity (DO), both of which are associated with symptoms of overactive bladder (OAB), such as urinary frequency and urgency [75–77]. Hormonal changes during pregnancy, particularly elevated levels of progesterone and relaxin, lead to relaxation of smooth muscles and connective tissue, which may contribute to pelvic floor dysfunction (PFD), a significant risk factor for OAB [78, 79]. Additionally, mechanical pressure and injury to the pelvic floor during labor can lead to long-term pelvic floor dysfunction, further impairing bladder control and promoting the development of OAB [80]. Moreover, post-menopausal estrogen deficiency can induce atrophic alterations in the urogenital tract, leading to increased urinary frequency, urgency, nocturia, incontinence, and recurrent infections. These factors collectively contribute to the higher incidence rate observed among women [81, 82].

The relationship between obesity and OAB remains controversial. A meta-analysis has found an association between BMI and an increased risk of OAB [83]. Similarly, another study identifies a higher BMI as a risk factor for developing OAB [84]. In contrast, a study involving 18,386 adult men failed to demonstrate an independent association between obesity and OAB [85]. Our results show that individuals who are overweight or obese have a higher prevalence of OAB. This phenomenon could be ascribed to the increased intra-abdominal pressure associated with a higher BMI, which can lead to nerve damage and pelvic floor dysfunction, thereby escalating the risk of OAB [86, 87]. Owing to abnormal metabolism in obese individuals, chronic pelvic ischemia and urinary tract damage are more likely to occur. Over time, these issues can lead to abnormal contractions of the detrusor muscle, thereby triggering OAB syndrome [88, 89]. Many epidemiological studies have shown a positive correlation between age and OAB [90–93]. Our results also indicate that the prevalence of OAB is higher in individuals aged 60 and above. With advancing age, inevitable atrophy of the genitourinary system occurs, rendering the elderly more susceptible to OAB [94]. Additionally, the elderly, being more susceptible to other chronic ailments such as Parkinson’s disease and diabetes, further exacerbate the likelihood of developing OAB [95, 96]. However, our study found a prevalence of 13.3% among individuals under 18, close to the reported 5–12% in the literature. This association could stem from the correlation between urinary system symptoms during childhood and the onset of OAB in adulthood. Additionally, children are prone to comorbidities such as anxiety, depression, and attention deficit issues, which may contribute to the manifestation of OAB symptoms. Moreover, there are genetic factors associated with OAB, which may explain the higher prevalence among individuals under 18 [97, 98].

This study also categorized countries into high-income countries (HICs), upper-middle-income countries (UMICs), and lower-middle-income countries (LMICs) based on World Bank income classifications to further explore the differences in the prevalence of OAB across varying economic backgrounds. Socioeconomic disparities have a significant impact on overall health, with lower socioeconomic status (SES) being positively correlated with increased risks of various diseases [99, 100]. A representative study of the adult population in the United States found that the prevalence of OAB was significantly lower in high-income groups compared to low-income groups [101]. Higher-income individuals typically have better access to health insurance and medical resources, allowing for timely diagnosis and treatment [102]. Additionally, healthier lifestyle factors, such as a balanced diet, regular physical activity, and greater health awareness, contribute to the reduction of chronic diseases, thus indirectly lowering the risk of OAB [103, 104]. Moreover, higher-income groups generally experience lower levels of environmental stress and benefit from stronger social support systems, which enhance their ability to cope with health challenges [105]. In contrast, in LMICs, the higher prevalence of comorbidities, such as diabetes and hypertension, significantly increases the risk of OAB [106].

In the six WHO regions covered in our study, meta-regression analysis indicated a correlation between the prevalence of OAB and regional distribution. The variations in prevalence across regions may be attributed to multiple factors, including demographic differences, cultural background, lifestyle changes, and racial disparities [27]. For instance, a study on racial differences in male OAB found that urgency urinary incontinence (UUI) and OAB without incontinence were most common among non-Hispanic Black men [107]. Another study revealed that the prevalence of OAB in African American men was significantly higher compared to Hispanic and Asian men [108]. In women, research has shown that Black women are 3.4-fold more likely to experience detrusor overactivity compared to their White counterparts [109]. Furthermore, while some studies report similar prevalence rates of lower urinary tract symptoms among Hispanic, African American, and White women, Asian women tend to have a relatively lower prevalence [108, 110, 111]. These racial and ethnic differences likely reflect variations in factors such as epigenetics, cultural background, physical activity, and dietary habits, which may collectively influence the development and progression of OAB, thus partially explaining the global disparities in its prevalence [112, 113].

In this analysis, eight different diagnostic tools were examined. A study examining OAB patients using the OABSS and IPSS questionnaires found no significant correlation between the two, which may help explain the considerable discrepancies in OAB prevalence rates observed across different diagnostic tools (although the potential impact of sample size differences on the results cannot be ruled out) [114]. It is important to note that each questionnaire tends to focus on distinct symptom dimensions or pathophysiological aspects. Additionally, one study suggests that the OAB-V8 tool exhibits high sensitivity; however, potential biases may arise when the physician’s knowledge of the scores is factored in, which could lead to an overestimation of its diagnostic effectiveness [115]. While some screening tools have undergone cross-cultural validation, demonstrating their applicability across various cultural contexts, it has been proposed that cultural and language differences may influence how patients report and interpret their symptoms, potentially affecting the accuracy of the assessments [66, 116–118]. These uncertainties likely contribute to the observed biases and variations in OAB prevalence rates across different diagnostic tools.

## Strengths and Limitations

This study excels in its comprehensive search strategy, dual-review process, and stringent selection criteria. Our systematic review exclusively encompassed studies targeting the general population to ensure the universality of our findings. Furthermore, we managed to synthesize the prevalence trends of OAB across WHO regions, WB regions, and nearly two decades based on available evidence, thereby enabling our systematic review and meta-analysis to provide a broad perspective on OAB prevalence. To our understanding, this marks the inaugural creation of a globally specific OAB prevalence and the exploration of its trends through a systematic review and meta-analysis, providing invaluable insights into the global burden of OAB.

However, we must acknowledge some inherent limitations. Despite the unified definition, there remains significant heterogeneity among the included studies. First, the etiology of OAB is multifactorial, with notable heterogeneity observed among different populations. These high-risk factors include advanced age, gender, obesity, gastrointestinal diseases, ethnicity, nerve damage, and urinary microbiota [52, 72, 119–121]. Second, the etiology of OAB remains incompletely understood, and there may be significant heterogeneity in symptoms among patients that are challenging to completely eliminate [122]. Additionally, despite conducting subgroup analyses based on the characteristics of included studies, heterogeneity among studies was not entirely eliminated, potentially impacting the accuracy of the meta-analysis results. Moreover, the publication year of the studies may not correspond to the exact year the population were studied, which could introduce temporal variations and affect the comparability of findings.

In summary, while this study offers valuable insights into the global prevalence and trends of OAB, it is essential to consider limitations such as heterogeneity and regional disparities when interpreting these findings. Future research should adopt standardized methods and broader geographical representation to enhance our comprehension of the global burden associated with OAB.

## Conclusion

This study underscores the substantial public health challenge presented by OAB on a global scale. Primarily, obesity, gender, and age emerge as predisposing factors for OAB. Over the past two decades, there has been a rising trend in OAB prevalence. Given the constraints of the studies, there is an urgent call for additional high-quality epidemiological research, especially in middle- to low-income countries, to delve deeper into the prevalence of OAB.

## Figures and Tables

**Fig. 3:**
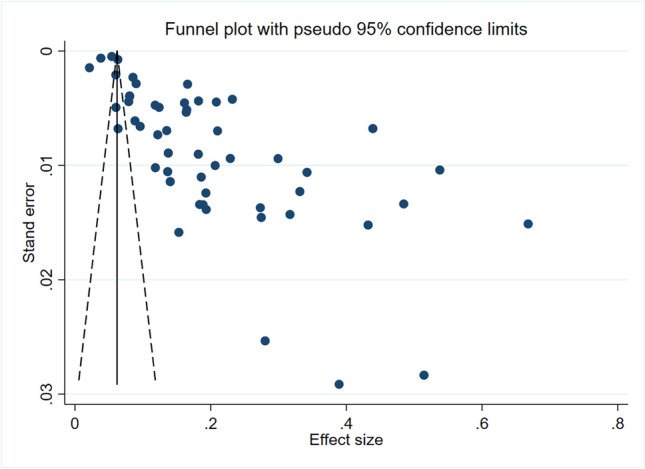
Funnel plot of meta-analysis on the prevalence of OAB

**Fig. 4:**
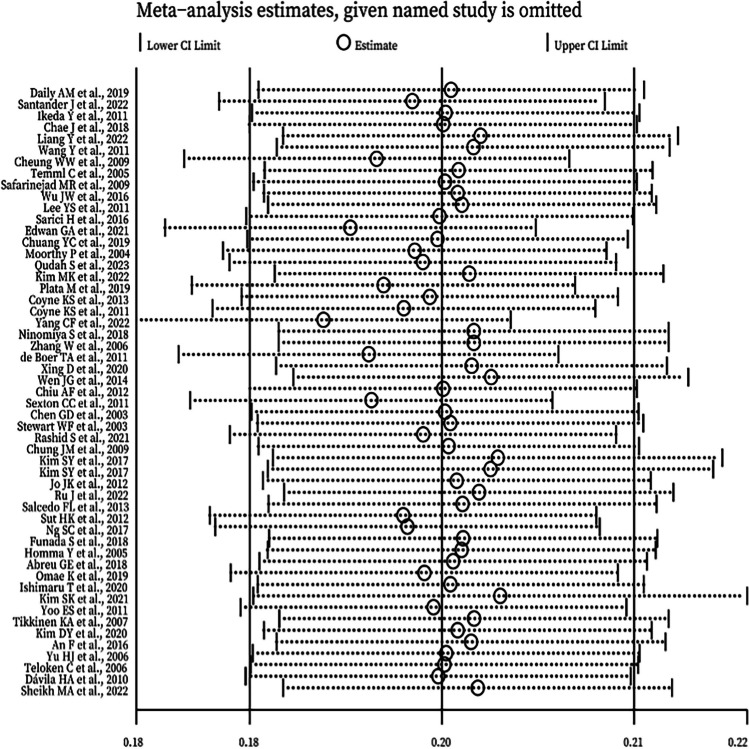
Leave-one-out sensitivity analysis

**Table 3.**
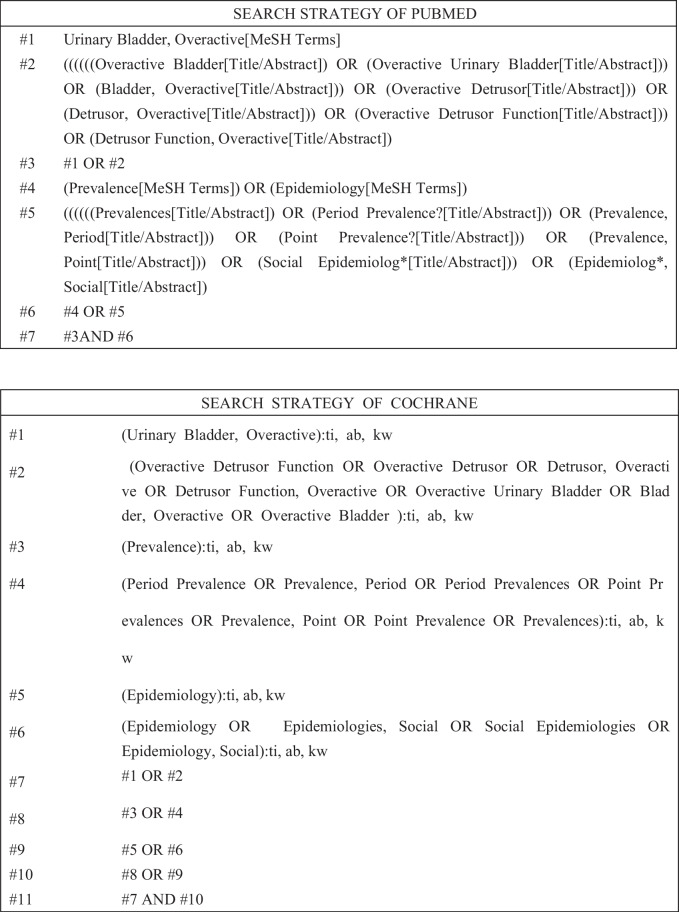

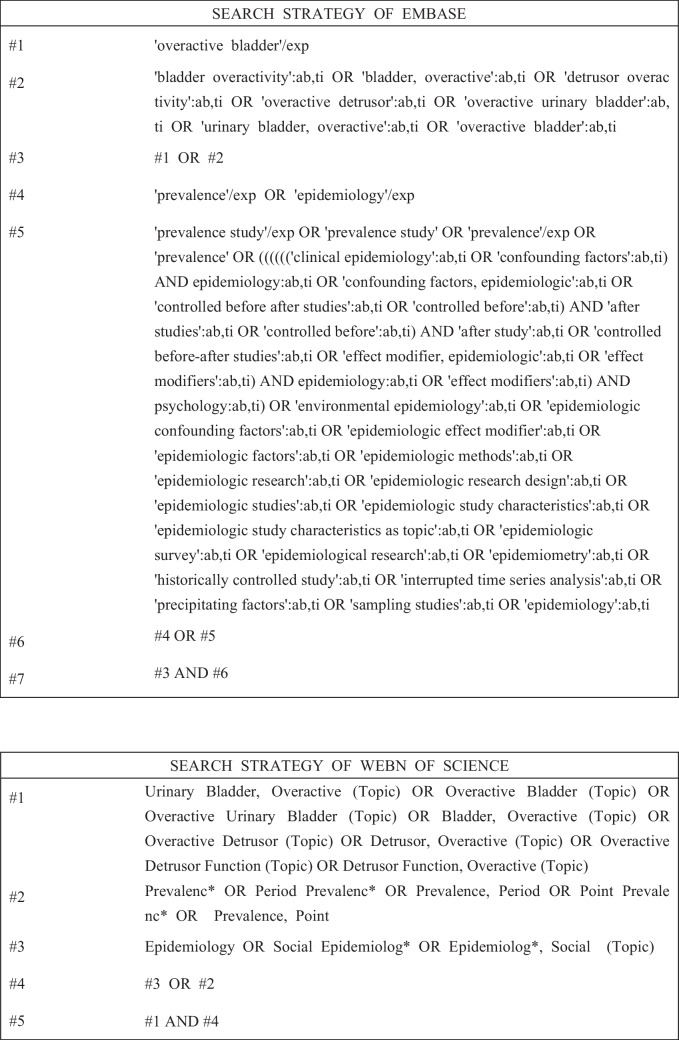
Search strategy

**Fig. 5:**
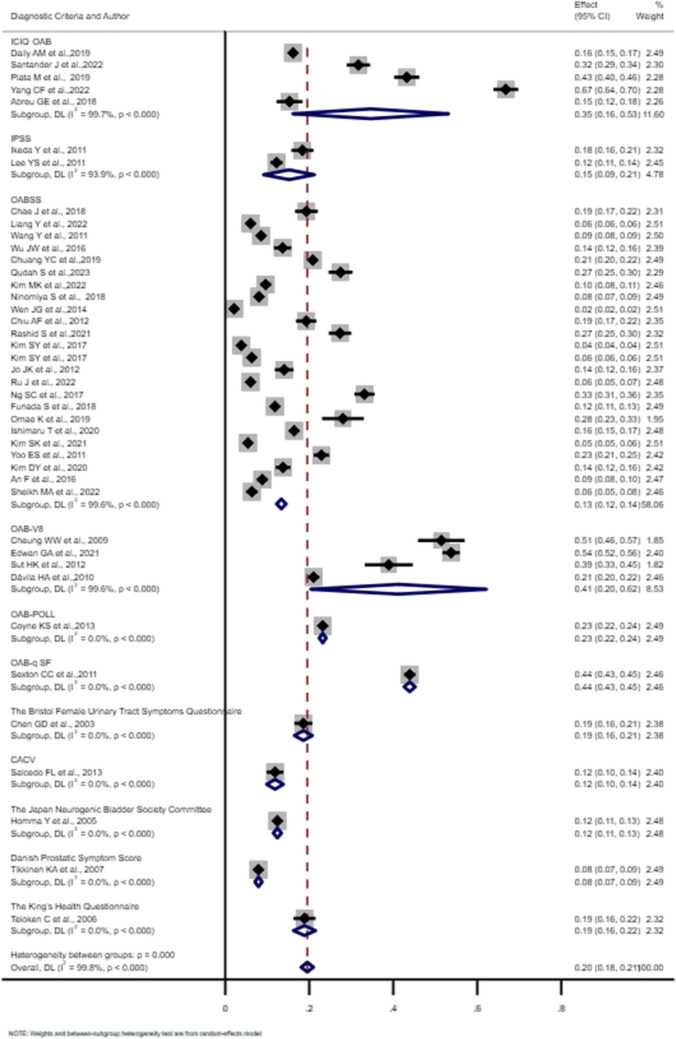
Prevalence across different measurement tools

## Data Availability

The data used in this study are publicly available from [specify the sources, e.g., PubMed, Embase, Cochrane, Web of Science]. These datasets are freely accessible and can be downloaded by any interested party.

## Appendix

Figures 3, 4

**Table 4:** Risk of bias question and total scores

Author (ref)	Q1	Q2	Q3	Q4	Q5	Q6	Q7	Q8	Q9	Q10	Q11	Total score
Daily AM et al. 2019 [14]	1	1	1	1	1	1	1	1	0	1	0	9
Santander J et al. 2022 [15]	1	1	0	1	1	1	1	1	0	1	0	8
Ikeda Y et al. 2011 [16]	1	1	1	1	1	1	1	1	0	1	0	9
Chae J et al. 2018 [17]	1	1	1	1	1	1	0	0	0	1	0	7
Liang Y et al. 2022 [18]	1	1	1	1	1	1	1	1	0	1	0	9
Wang Y et al. 2011 [19]	1	1	1	1	1	1	0	0	0	1	0	7
Cheung WW et al. 2009 [20]	1	1	0	1	1	1	0	0	0	1	0	6
Temml C et al. 2005 [21]	1	1	0	1	1	1	0	0	0	1	0	6
Safarinejad MR et al. 2009 [22]	1	1	1	1	1	1	1	1	0	1	0	9
Wu JW et al. 2016 [23]	1	1	1	1	1	1	1	1	0	1	0	9
Lee YS et al. 2011 [24]	1	1	1	1	1	1	0	0	0	1	0	7
Sarici H et al. 2016 [25]	1	1	1	1	1	1	1	1	0	1	0	9
AI Edwan G et al. 2021 [26]	1	1	1	1	1	1	1	1	0	1	0	9
Chuang YC et al. 2019 [27]	1	1	0	1	1	1	0	0	0	1	0	6
Moorthy P et al. 2004 [28]	1	1	1	1	1	1	1	1	0	1	0	9
Qudah S et al. 2024 [29]	1	1	0	1	1	1	1	1	0	1	0	8
Kim MK et al. 2022 [31]	1	1	0	1	1	1	0	0	0	1	0	6
Plata M et al. 2019 [32]	1	1	1	1	1	1	1	1	0	1	0	9
Coyne KS et al. 2013 [10]	1	1	1	1	1	1	1	1	0	1	0	9
Coyne KS et al. 2011 [33]	1	1	1	1	1	1	0	0	0	1	0	7
Yang CF et al. 2022 [34]	1	1	0	1	1	1	0	0	0	1	0	6
Ninomiya S et al. 2018 [35]	1	1	0	1	1	1	0	0	0	1	0	6
Zhang W et al. 2006 [36]	1	1	1	1	1	1	0	0	0	1	0	7
de Boer TA et al. 2011 [37]	1	1	0	1	1	1	0	0	0	1	0	6
Xing D et al. 2020 [38]	1	1	1	1	1	1	0	0	0	1	0	7
Wen JG et al. 2014 [39]	1	1	0	1	1	1	1	1	0	1	0	8
Chiu AF et al. 2012 [40]	1	1	1	1	1	1	1	1	0	1	0	9
Sexton CC et al. 2011 [41]	1	1	1	1	1	1	0	1	0	1	0	8
Chen GD et al. 2003 [42]	1	1	1	1	1	1	0	0	0	1	0	7
Stewart WF et al. 2003 [4]	1	1	0	1	1	1	1	1	0	1	0	8
Rashid S et al. 2021 [43]	1	1	1	1	1	1	1	1	0	1	0	9
Chung JM et al. 2009 [44]	1	1	1	1	1	1	0	0	0	1	0	7
Kim SY et al. 2017 [45]	1	1	1	1	1	1	0	0	0	1	0	7
Kim SY et al. 2017 [46]	1	1	1	1	1	1	0	0	0	1	0	7
Jo JK et al. 2012 [47]	1	1	1	1	1	1	0	0	0	1	0	7
Ru J et al. 2022 [48]	1	1	1	1	1	1	0	0	0	1	0	7
Salcedo FL et al. 2013 [49]	1	1	1	1	1	1	0	0	0	1	0	7
Sut HK et al. 2012 [50]	1	1	1	1	1	1	1	1	0	1	0	9
Ng SC et al. 2017 [51]	1	1	1	1	1	1	0	0	0	1	0	7
Funada S et al. 2018 [52]	1	1	1	1	1	1	1	1	0	1	0	9
Homma Y et al. 2005 [53]	1	1	0	1	1	1	0	0	0	1	0	6
Abreu GE et al. 2018 [54]	1	1	1	1	1	1	1	1	0	1	0	9
Omae K et al. 2019 [55]	1	1	1	1	1	1	1	1	0	1	0	9
Ishimaru T et al. 2020 [56]	1	1	1	1	1	1	0	0	0	1	0	7
Kim SK et al. 2021 [57]	1	1	1	1	1	1	0	0	0	1	0	7
Yoo ES et al. 2011 [58]	1	1	1	1	1	1	1	1	0	1	0	9
Tikkinen KA et al. 2007 [59]	1	1	1	1	1	1	1	1	0	1	0	9
Kim DY et al. 2020 [60]	1	1	0	1	1	1	1	1	0	1	0	8
An F et al. 2016 [9]	1	1	1	1	1	1	1	1	0	1	0	9
Yu HJ et al. 2006 [61]	1	1	1	1	1	1	1	1	0	1	0	9
Teloken C et al. 2006 [62]	1	1	1	1	1	1	1	1	0	1	0	9
Dávila HA et al. 2010 [63]	1	1	1	1	1	1	0	0	0	1	0	7
Sheikh MA et al. 2022 [30]	1	1	1	1	1	1	0	0	0	1	0	7
